# PIN3 duplication may be partially responsible for *TP53* haploinsufficiency

**DOI:** 10.1186/1471-2407-14-669

**Published:** 2014-09-15

**Authors:** Marta Winiecka-Klimek, Malgorzata Szybka, Piotr Rieske, Sylwester Piaskowski, Michal Bienkowski, Maciej Walczak, Marcin Pacholczyk, Michal Rostkowski, Jolanta Zieba, Mateusz Banaszczyk, Krystyna Hulas-Bigoszewska, Joanna Peciak, Rafal Pawliczak, Ewelina Stoczynska-Fidelus

**Affiliations:** Department of Tumor Biology, Medical University of Lodz, Zeligowskiego 7/9, 90–752 Lodz, Poland; Department of Microbiology and Medical Laboratory Immunology, Medical University of Lodz, Pomorska 251, 92-213 Lodz, Poland; Department of Molecular Pathology and Neuropathology, Chair of Oncology, Medical University of Lodz, Czechoslowacka 8/10, 92-216 Lodz, Poland; Institute of Automatic Control, Silesian University of Technology, Akademicka 2A, 44-100 Gliwice, Poland; Institute of Applied Radiation Chemistry, Faculty of Chemistry, Lodz University of Technology, Zeromskiego 116, 90-924 Lodz, Poland; Department of Immunopathology, Medical University of Lodz, Zeligowskiego 7/9, 90-752 Lodz, Poland

**Keywords:** *TP53*, *PIN3*, *Heterozygous mutation*, *Haploinsufficiency*, *G-quadruplex*, *Cancer cell lines*

## Abstract

**Background:**

Previously we have suggested that cancer cells develop a mechanism(s) which allows for either: silencing of the wild-type *TP53* transcription, degradation of the wild-type *TP53* mRNA, or selective overproduction of the mutated *TP53* mRNA, which is the subject of this article. Sequencing of *TP53* on the respective cDNA and DNA templates from tumor samples were found to give discordant results. DNA analysis showed a pattern of heterozygous mutations, whereas the analysis of cDNA demonstrated the mutated template only. We hypothesized that different *TP53* gene expression levels of each allele may be caused by the polymorphism within intron 3 (PIN3). The aim of this study was to test if one of the polymorphic variants of PIN3 (A1 or A2) in the heterozygotes is associated with a higher *TP53* expression, and therefore, responsible for the haploinsufficiency phenomenon.

**Methods:**

250 tumor samples were tested. To analyze the involvement of PIN3 polymorphic variant (A1 or A2) on *TP53* mRNA expression regulation, bacterial subcloning combined with sequencing analyses, dual luciferase reporter assays and bioinformatic analysis were performed.

**Results:**

Haplotype analysis showed the predominance of the mutated template during the cDNA sequencing in all samples showing a heterozygous *TP53* mutation and PIN3 heterozygosity. Out of 30 samples (from the total of 250 tested samples) which carried *TP53* mutations and had a bias in allelic expression 6 were heterozygous for the A1/A2 polymorphism, and all 6 (p = 0.04) samples carried the mutation within the PIN3 longer allele (A2). Reporter assays revealed higher luciferase activity in cells transfected with the plasmid containing A2 construct than A1 and control. A2/A1 ratio ranged from 1.16 for AD293 cell line (p = 0.019) to 1.59 for SW962 cell line (p = 0.0019). Moreover, bioinformatic analyses showed that PIN3 duplication stabilized secondary DNA structures – G-quadruplexes.

**Conclusion:**

*TP53* alleles are not equivalent for their impact on the regulation of expression of *TP53* mRNA. Therefore, in PIN3-heterozygous cases a single *TP53* mutation of the longer allele might sufficiently destabilize its function. Secondary DNA structures such as quadruplexes can also play a role in PIN3-dependent TP53 haploinsufficiency.

**Electronic supplementary material:**

The online version of this article (doi:10.1186/1471-2407-14-669) contains supplementary material, which is available to authorized users.

## Background

TP53 is one of the most prominent suppressor proteins and the respective gene is the most thoroughly studied one. Typically, tumor suppressor genes show either homo- or hemizygous mutations, but *TP53* is an exception in this aspect [1]. We have already suggested that the dominant negative effect and gain of function are supported by an unknown mechanism causing higher expression of the mutated allele (in comparison to the normal allele) in cells with a heterozygous mutation [2]. Here, we made a further insight into the influence of PIN3 polymorphism on the regulation of *TP53* expression.

We have already reported the differences in the results of sequencing of the *TP53* gene between the analyses performed on DNA and those performed on the respective cDNA [3]. The former presented the heterozygous pattern, whereas the latter demonstrated the mutated template only. This observation was an incentive to the search for an unknown haploinsufficiency mechanism. We hypothesized that the differences in the expression levels between alleles may be an effect of polymorphisms or epigenetic changes.

Albeit relatively infrequent, *TP53* polymorphisms may be important from the perspective of susceptibility to various cancers. PIN3 and codon 72 polymorphisms are the two most frequently observed. Codon 72 polymorphism, leading to arginine-proline substitution, and thus, affecting the structure of the resulting protein [4], occurs within a proline-rich region, which controls growth suppression and apoptosis [5, 6]. It has been reported that Arg/Arg homozygotes are more efficient in apoptosis induction than Pro/Pro homozygotes, which, in turn, play a role in cell cycle arrest in G1 phase [7, 8]. Codon 72 polymorphism has been reported as associated with breast, lung and bladder cancer susceptibility [9–11].

Polymorphism in *TP53* intron 3 (PIN3) is a 16 bp duplication (5’-ACCTGGAGGGCTGGGG-3’). The allele with only one repetition of 16 bp at the PIN3 site is usually marked as A1 or N (non-duplicated), the other (with 2 repetitions) is marked as A2 or D (duplicated). PIN3 is also responsible for the unique DNA conformer construction – a *G-quadruplex* structure, which overlaps intron 3 [12].

Since PIN3 is related to higher cancer susceptibility (including breast and colorectal) [13–15], we hypothesized that the insertion may cause different expression of each allele. Both exon 3 (22 bp) and intron 3 (93 bp) of the *TP53* gene are relatively short, so an increase of intron 3 length by 17% (16 bp) may even influence the protein function. As reported by Mergny *et al*., the primary nucleotide sequence composition of tetramolecular structures can affect and change melting temperature and association constant values [16], which influences both G-quadruplex formation and stability, further affecting gene expression and protein function [16]. Marcel *et al.* described the importance of intronic G-quadruplexes in the process of *TP53* alternative splicing [12]. Formation of the mentioned structures interferes with the excision of intron 2, which has an alternative translation start site, resulting in a Δ40p53 isoform [12], which, when expressed in excess, exerts a negative effect on the wild-type protein [17]. Finally, Uhlemann *et al*. suggested that not only the regulatory elements, but also the areas between them affect the gene expression [18]. They reported that the variation of TA repeats polymorphism upstream the promoter of the *gp91*^*phox*^ results in differences in the activation of *gp91*^*phox*^ promoter [18].

Gemignani *et al*. reported that the shorter allele (A1) provides higher expression than does the longer allele (A2) [14]. Thus, our hypothesis could be tested for the selected group of cases (heterozygotes A1/A2). In these cases, the mutation of the allele allowing for the higher *TP53* expression overrides the normal TP53 activity (through definite predominance of the mutated mRNA) despite the retention of the wild-type allele at the DNA level (haploinsufficiency). This lead to the aim of this study – to investigate if PIN3 polymorphism play a role in TP53 haploinsufficiency. Undoubtedly, behind the haploinsufficiency phenomenon there is an unidentified mechanism, which probably modulates *TP53* mRNA expression.

## Methods

### Material

The study included human cancer cell lines, cultured cells and tumor samples. The commercially available human cell lines were obtained from American Type Culture Collection (ATCC, Manassas, USA – SW962) and from Stratagene (California, USA – AD293). For this study the *TP53* sequencing results from more than 250 tumor samples were analyzed. We have reviewed the sequencing results for 307 samples (partially previously published [2, 3, 19, 20], partially performed specifically for this analysis). Since a portion of the archival material was no longer available, PIN3 and codon 72 analysis was possible in 250 cases, among which there were 45 cases with missense mutations (Table 1), which were subjected to further analysis. All samples were collected using the protocols approved by the Bioethical Committee of the Medical University of Lodz (Approval No. RNN/9/10/KE and No. RNN/53/08/KE). Written informed consent was obtained from all patients and their data were processed and stored according to the principles expressed in the Declaration of Helsinki.

**Table 1:** **Results of DNA and cDNA sequencing analysis in samples with** ***TP53*** **mutations** [2, 3, 19, 20]

No.	Diagnosis	***TP53***mutations			PIN3 status	72 codon status
		Location/type	cDNA	DNA		
*TP53* mutations; cases showing discrepancies between cDNA and DNA						
1	Glioblastoma	MT1 175; CGC > CAC; Arg > His	MT1 > MT2	MT1 = MT2	A1/A2	C/G
		MT2 282; CGG > TGG; Arg > Trp				
2	Glioblastoma	237; ATG > ATA; Met > Ile	MT	WT > MT	A1/A2	C/G
3	Glioblastoma	273; CGT > CAT; Arg > His	MT	WT > MT	A1	C
4	Glioblastoma	234; TAC > CAC; Tyr > His	MT	WT > MT	A1	G
5	Glioblastoma	273; CGT > TGT; Arg > Cys	MT	WT > MT	A1	G
6	Glioblastoma	190; CCT > TCT; Pro > Ser	MT	WT = MT	A1	G
7	Glioblastoma	152; CCG > CTG; Pro > Leu	MT	WT = MT	A2	C
8	Glioblastoma	273; CGT > TGT; Arg > Cys	MT	WT = MT	A2	G
9	Glioblastoma	237; ATG > ATA; Met > Ile	MT	MT > WT	A1	C
10	Glioblastoma	161; GCC > ACC; Ala > Thr	MT	MT > WT	A2	C/G
11	Soft tissue sarcoma	248; CGG > CAG; Arg > Gln	MT	WT	A1	G
12	Soft tissue sarcoma	273; CGT > TGT; Arg > Cys	MT	WT = MT	A1	G
13	Soft tissue sarcoma	216; GTG > ATG; Val > Met	MT	MT > WT	A1/A2	C/G
14	Colorectal cancer	173; GTG > ATG; Val > Met	MT	MT > WT	A1	G
15	Colorectal cancer	248; CGG > TGG; Arg > Trp	MT	WT	A1	C/G
16	Colorectal cancer	175; CGC > CAC; Arg > His	MT	WT = MT	A1	G
17	Colorectal cancer	273; CGT > CAT; Arg > His	MT > WT	WT = MT	A1	G
18	Colorectal cancer	285; GAG > AAG; Glu > Lys	MT > WT	WT	A1	G
19	Colorectal cancer	245; GGC > AGC; Gly > Ser	MT	WT > MT	A2	C
20	Colorectal cancer	248; CGG > CAG; Arg > Gln	MT	WT > MT	A1/A2	C/G
21	Colorectal cancer	273; CGT > CAT; Arg > His	MT > WT	WT > MT	A1/A2	C/G
22	Colorectal cancer	282; CGG > TGG; Arg > Trp	MT	WT > MT	A1	G
23	Colorectal cancer	245; GGC > AGC; Gly > Ser	MT = WT	WT > MT	A1	G
24	Colorectal cancer	216; GTG > ATG; Val > Met	MT > WT	WT = MT	A1	G
25	Colorectal cancer	245; GGC > AGC; Gly > Ser	MT = WT	WT > MT	A1	G
26	Colorectal cancer	175; CGC > CAC; Arg > His	MT	WT = MT	A1	G
27	Colorectal cancer	175; CGC > CAC; Arg > His	MT	MT > WT	A2	C/G
28	Leukemia (AML)	216; GTG > ATG; Val > Met	MT	WT	A1	C/G
29	Leukemia (AML)	267; CGG > GGG; Arg > Gly	MT = WT	WT	A1	C/G
30	Prostate cancer	239; AAC > GAC; Asn > Asp	MT	WT = MT	A1/A2	C/G
*TP53* mutations; no discrepancies between DNA and cDNA sequencing						
31	Glioblastoma	214; AGT > AAT; Ser > Asn	WT = MT	WT = MT	A1	G
32	Glioblastoma	282; CGG > TGG; Arg > Trp	WT = MT	WT = MT	A1	G
33	Astrocytoma	179; CAT > GAT; His > Asp	WT = MT	WT = MT	A1	C/G
34	Glioblastoma	267; CGG > TGG; Pro > Trp	WT = MT	WT = MT	A1	C/G
35	Glioblastoma	173; GTG > TTG; Val > Leu	WT = MT	WT = MT	A1	G
36	Glioblastoma	273; CGT > TGT; Arg > Cys	WT = MT	WT = MT	A1	G
37	Glioblastoma	190; CCT > CTT; Pro > Leu	WT = MT	WT = MT	A2	C
38	Glioblastoma	145; CTG > CAG; Leu > Gln	MT	MT	A1	C
39	Soft tissue sarcoma	273; CGT > TGT; Arg > Cys	WT = MT	WT = MT	A1	G
40	Soft tissue sarcoma	215; AGT > AAA; Ser > Lys	MT > WT	MT > WT	A1	C/G
41	Soft tissue sarcoma	248; CGG > CAG; Arg > Gln	MT	MT	A1	G
42	Soft tissue sarcoma	173; GTG > TTG; Val > Leu	MT	MT	A1/A2	C/G
43	Colorectal cancer	273; CGT > CAT; Arg > His	WT = MT	WT = MT	A1	C
44	Colorectal cancer	134; TTT > CTT; Phe > Leu	WT = MT	WT = MT	A1	C/G
45	Colorectal cancer	175; CGC > CAC; Arg > His	WT = MT	WT = MT	A1	C/G

AML – acute myeloid leukemia; WT – wild-type template; MT – mutated template; A1, A2 – polymorphic variants of PIN3; C – cytosine; G – guanine.

### Cell culture

Cells were cultured in MEM or DMEM supplemented with 10% FBS (PAA, Linz, Austria) and antibiotics (penicillin/streptomycin/gentamicin; GIBCO, BRL, Paisley, Great Britain) in 5% CO_2_. Adherent cells were passaged with trypsin (GIBCO) before obtaining 70% confluence.

### DNA and RNA isolation

Total DNA and RNA were isolated from cell cultures and frozen tumor fragments (stored at -80°C) and peripheral blood leukocytes obtained from patients and healthy volunteers. The isolation was performed using AllPrep DNA/RNA Mini Kit (Qiagen, Hilden, Germany) according to the manufacturer's protocol. During RNA isolation DNase was used. Nucleic acid concentration was determined spectrophotometrically. 100 ng of total RNA was reverse transcribed into single-stranded cDNA using QuantiTect Rev. Transcription Kit (Qiagen) according to the manufacturer’s protocol.

### *TP53*DNA and cDNA sequencing

Exons 4 – 8 of *TP53* were sequenced in search of mutations. The primers used for the PCR amplification of *TP53* DNA and cDNA sequences and sequencing primers are listed in tables A1 and A2 (Additional file 1). *TP53* sequencing was performed using BigDye Seq kit v3.1 (Applied Biosystems, Foster City, CA, USA). The sequences were analyzed with the ABI 3130 genetic analyzer and DNA Sequencing Analysis Software (Applied Biosystems).

### Bacterial subcloning of a DNA fragment containing the *TP53*codon 72 and PIN3 from samples with a single heterozygous mutation

**Figure 1:**
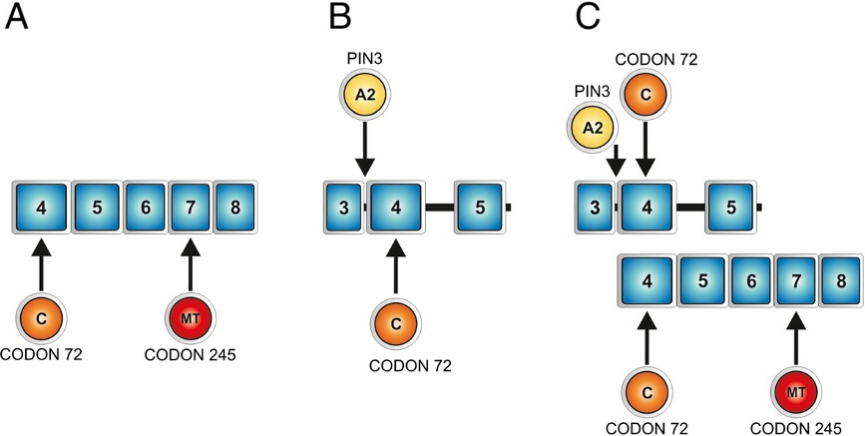
**A schematic diagram of the subcloning procedure. A**. Sequencing of *TP53* cDNA fragment containing exons 4 – 8, that specify which allele with cytosine or guanine in codon 72 (exon 4) is mutated within exons 5 – 8. **B**. The samples containing an exonic mutation and codon 72 heterozygosity were subjected to bacterial subcloning. A fragment of *TP53* gene comprising intron 3 and codon 72 from the selected samples were cloned into a bacterial vector and sequenced. Such an analysis allowed for the sequencing of each allele separately, and therefore, for the detection of haplotypes (*i.e.* which codon 72 variant co-localized with which PIN3 variant). **C**. Combination of these results allows to infer which PIN3 allele (A1 or A2) is the mutated one.

**Figure 2:**
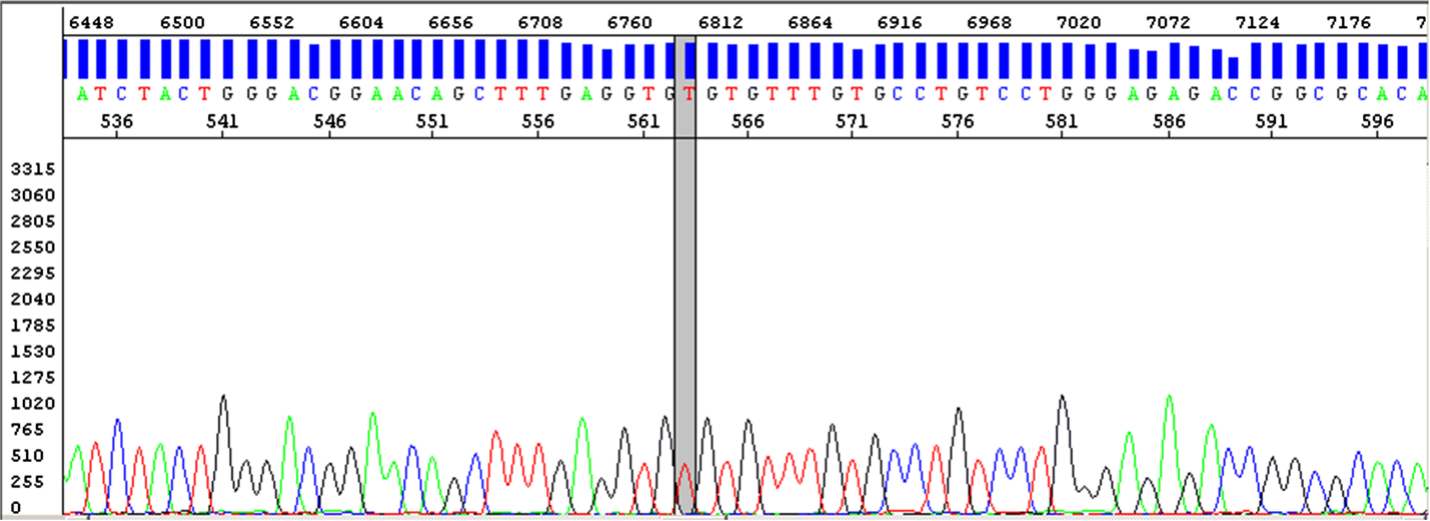
**The result of capillary sequencing of the** ***TP53*** **exon 8 fragment with the designated R273C mutation (the line marks the mutated nucleotide).**

Bacterial subcloning was performed in order to determine which allele (A1 or A2) is preferentially mutated in samples with a heterozygous *TP53* mutation. Since PIN3 is an intronic polymorphism, it may only be analyzed at the DNA level. Therefore, a direct analysis would require the subcloning of about 3 thousand nucleotide-long sequence (the distance between intron 3 and exon 8) into bacterial vectors. To avoid the technical inconveniences we performed an indirect analysis using another polymorphic site at codon 72 (Figure 1). Such an analysis was only possible for the samples with heterozygous *TP53* mutation, PIN3 A1/A2 heterozygosity, codon 72 C/G heterozygosity and predominance of the mutated template during the cDNA sequencing. Firstly, cDNA sequencing of exons 4 – 8 was used to determine which allele (at codon 72) is mutated (Figure 2), since the predominance of the mutated allele would be also observed here. Subsequently, the bacterial subcloning of DNA fragments containing intron 3 and exon 4 (and thus, both polymorphic sites) from samples with a single heterozygous mutation was performed. It allowed for the sequencing of each allele separately, and therefore, for the detection of haplotypes (*i.e.* which codon 72 variant co-localized with which PIN3 variant). From these observations it may be easily inferred which PIN3 variant was mutated; *e.g.* if cDNA analysis revealed that the mutation co-localized with cytosine in codon 72 and subcloning showed that codon 72 cytosine co-localized with the longer PIN3 variant, we may conclude that the mutation occurred within the allele with A2 PIN3 variant. As the vector for cloning pUC19 plasmid was used. The *TP53* DNA fragment was amplified by PCR using the primers complementary to the target DNA with additional nucleotides at the 5’ ends to facilitate digestion (Table A3, Additional file 1). Both the PCR product and the vector were digested with restriction enzymes BamHI and HindIII (Fermentas, Thermo Fisher Scientific, Waltham, USA) in two separate reactions according to the manufacturer's protocol. 1-sample proportion test with continuity correction was used to assess the probability of the observed allele distribution.

### Plasmids construction

Generation of the reporter assay plasmids was performed by inserting DNA fragments comprising the intron 3 sequence of the *TP53* gene, obtained with PCR on the heterozygous template. The PCR products were separated with 2% agarose gel electrophoresis and DNA was extracted from the gel using AxyPrep DNA Gel Extraction Kit (Axygen, Corning, New York, USA). The fragments of 174 and 190 bp were chosen for insertion into pTKLuc + reporter plasmid (ATCC). Both the plasmid and the inserts with the sequence of interest were digested with BamHI and HindIII restriction enzymes (New England Biolabs, NEB, Ipswich, USA) in two separate reactions according to the manufacturer's protocol. The products were purified by QIAquick PCR Purification Kit (Qiagen) and ligated for 30 min with T4Quick Ligase (NEB) according to the manufacturer’s protocol. The ligation products were used for the transformation of *E. coli* NEB Turbo competent cells (NEB). The plasmids from the selected clones were sequenced to distinguish different polymorphic variants (construct A1 from construct A2) and to confirm the correct insertion of both constructs.

### Dual luciferase reporter assay

Two cell lines with different characteristics were used for the transfection and the subsequent dual luciferase reporter assay: AD293 and SW962. AD293 (which is commonly used for dual luciferase reporter assays [21, 22]), contained only wild-type *TP53* and constituted the normal sample. SW962 was chosen as a cell line with heterozygous mutation of *TP53*. For the reporter assay cells were seeded in 6-well plates (2.5x10^5^ cells/well) 24 h prior to the transfection. Cells were cotransfected with 0.25 μg of specific pTKLuc + reporter plasmid (ATCC; containing Firefly luciferase and either A1, A2 or no insert) and 0.25 μg of pGL4.74 control vector (containing Renilla luciferase; Promega, Madison, USA) using Lipofectamine Plus Reagent (Invitrogen, Carlsbad, USA) in OptiMEM (GIBCO) without antibiotics. 24 h posttransfection the cells were lysed using Passive Lysis Buffer (Promega). The lysates were analyzed by measurement of luciferase activity (Firefly and Renilla) using Dual-Luciferase Reporter Assay System (Promega) according to the manufacturer’s protocol on TD20/20 Luminometer (Promega). For AD293 cell line – 11 and for SW962 cell line – 8 independent experiments were performed. The ratios of luciferase activity were normalized (Fluc value/Rluc value). The ratios for the constructs were subsequently normalized to the control samples (C, transfected with the plasmid without inserts). The results are presented as mean ratios from independent experiments for each cell line, compared to mean ratio obtained for the control sample (Table A4, Additional file 1). The statistical analysis was performed using Mann–Whitney *U*-test.

### Bioinformatic analysis

The sequence of *TP53* intron 3, potentially capable of forming G-quadruplex structure, was obtained from IARC TP53 Database (Table A5, see Additional file 1) [23]. To predict the secondary DNA structure of *TP53* intron 3, RNAfold from ViennaRNA package version 2.1.6 with DNA energy parameters was used [24]. The RNAfold predicts secondary DNA structure through energy minimization using dynamic programming [25]. The default minimum free energy algorithm which yields the single optimal structure was used.

## Results

### *TP53*DNA and cDNA sequencing

Among the 307 samples there were 97 gliomas, 94 soft tissue sarcomas, 31 colorectal cancers, 20 prostate cancers, 23 acute myeloid leukemias and 42 invasive breast duct carcinomas. Alterations of the *TP53* gene were detected in 64 (20.8%) tumors (Table A6, see Additional file 1); a high number of *TP53* mutated samples were found in colorectal cancer (19/31; 61.3%) and in glioma (25/97; 25.8%). *TP53* mutations were also detected in 19% (8/42) of invasive breast duct carcinoma, 9.6% of soft tissue sarcoma (9/94), 8.7% of acute myeloid leukemia (2/23) and 5% of prostate cancer cases (1/20). For further analyses the 45 cases with missense mutations were used (Table 1). Within this group 15 showed consistent results of DNA and cDNA sequencing, while 30 cases showed differences between them (Table 1).

### PIN3 polymorphism analysis for cases with *TP53*mutation

PIN3 polymorphism status was identified with sequencing (Table 1). Among the cases with a *TP53* mutation and no differences between DNA and cDNA, 13 were recognized as A1/A1 homozygotes, 1 as A2/A2 homozygote and 1 as heterozygote. On the other hand, among the cases with differences between DNA and cDNA, there were 19 A1/A1 homozygotes, 5 A2/A2 homozygotes and 6 heterozygotes, which constituted the starting point for further analysis aiming to determine the haplotype of the tested alterations.

### Bacterial subcloning of a DNA fragment containing the *TP53*codon 72 and PIN3 from samples with a single heterozygous mutation

The sequencing of the obtained clones revealed that the mutation co-localized with the longer PIN3 variant (A2) in all six cases (p = 0.04) (Table 2) as well as with cytosine at codon 72 (in 5 out of 6 cases), both of which are the less common variants [13, 14].

**Table 2:** **Results of DNA and cDNA sequencing combined with the results of bacterial subcloning analyses**

Number of sample	Diagnosis	***TP53***mutations			PIN3	Codon 72 (DNA)	Codon 72 (cDNA)	Subcloning results			
		Type	cDNA	DNA				Colonies	PIN3	Codon 72	Conclusions
1	Glioblastoma	MT1 175;	MT1 > MT2	MT1 = MT2	A1/A2	C/G	C	L1-2	A1	G	A2 MT1
		CGC > CAC									
		Arg > His									
		MT2 282									
		CGG > TGG									
		Arg > Trp									
2	Glioblastoma	237; ATG > ATA Met > Ile	MT	WT > MT	A1/A2	C/G	C	L2-2	A1	G	A2 MT
13	Soft tissue sarcoma	216; GTG > ATG Val > Met	MT	MT > WT	A1/A2	C/G	C	L3-1 L3-2	A2	C	A2 MT
20	Colorectal cancer	248; CGG > CAG Arg > Gln	MT	WT > MT	A1/A2	C/G	G	L4-2	A1	C	A2 MT
21	Colorectal cancer	273; CGT > CAT Arg > His	MT > WT	WT > MT	A1/A2	C/G	C	L5-1 L5-2	A2	C	A2 MT
30	Prostate cancer	239; AAC > GAC Asn > Asp	MT	MT = WT	A1/A2	C/G	C	L6-2	A2	C	A2 MT

MT – mutated template; WT – wild-type template; A1, A2 - polymorphic variants of PIN3; C – cytosine; G – guanine.

### Dual luciferase assay

**Figure 3:**
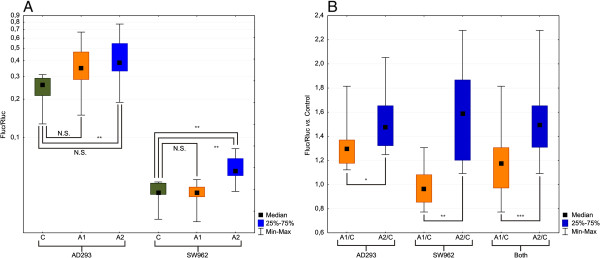
Luciferase activity in cells transfected with control, A1 and A2 constructs (* p < 0,05; ** p < 0,01; *** p < 0,001). A. Normalized luciferase activity depending on cell line and transfecting construct. **B**. Normalized luciferase activity ratios of A1/A2 constructs versus control.

Genetic reporter assay confirmed that in all samples the transfection with construct A2 resulted in the higher luciferase expression than did the transfection with construct A1 (Figure 3; Table A4, Additional file 1). The difference between the luciferase activity of the control sample and A1 variant was marginal and not statistically significant (A1/C = 1.32; p = 0.056 for AD293; A1/C = 0.98; p = 0.645 for SW962), while samples transfected with A2 variant showed significantly higher luciferase activity than the control sample (A2/C =1.53; p = 0.008 for AD293; A2/C = 1.59; p = 0.001 for SW962). A2/A1 ratio ranged from 1.16 for AD293 (p = 0.019) to 1.59 for SW962 (p = 0.0019). The collective analysis for both cell lines proved the significance of the differences between the two variants (p = 0.00019). Normalized luciferase activities (Fluc/Rluc value) from independent experiments for each construct (A1 or A2) compared to normalized luciferase activities obtained for control sample are presented in Figure 3A. Graphs illustrate also comparison of ratios of A2 to A1 for each cell line separately and for both cell lines (Figure 3B).

### Bioinformatic analysis

**Figure 4:**
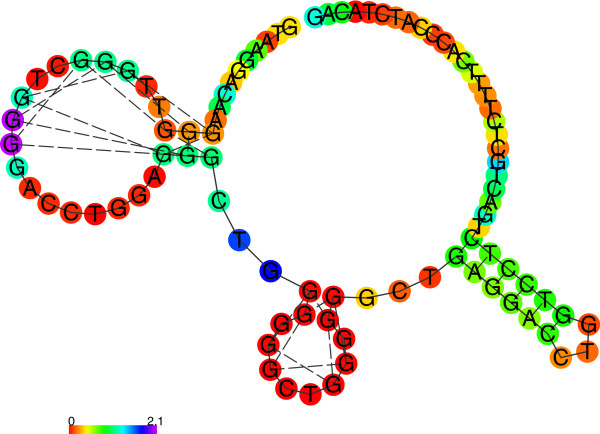
***TP53*** **intron 3 (A1) secondary structure prediction (in G-quadruplex prediction mode).**

**Figure 5:**
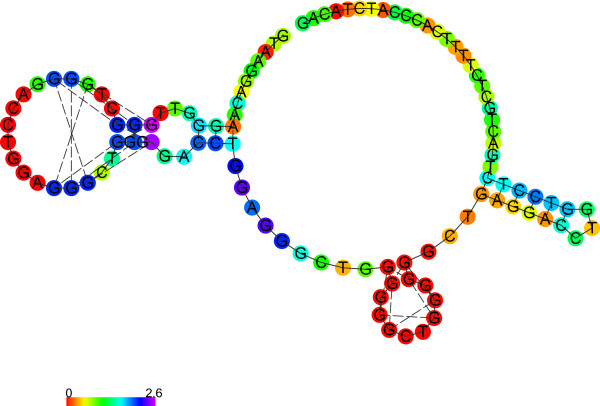
***TP53*** **intron 3 with 16 bp duplication (A2) secondary structure prediction (in G-quadruplex prediction mode).**

**Figure 6:**
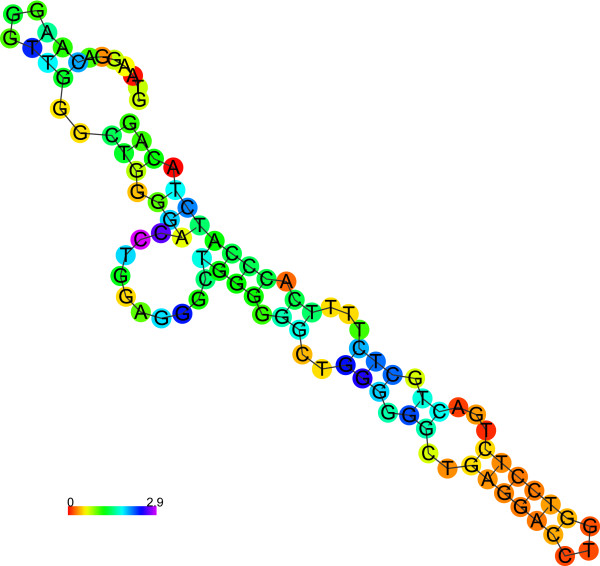
***TP53*** **intron 3 (A1) secondary structure prediction (in canonical structure prediction mode).**

**Figure 7:**
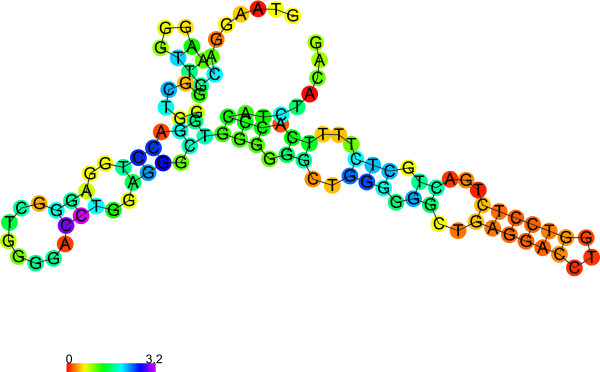
***TP53*** **intron 3 with 16 bp duplication (A2) secondary structure prediction (in canonical structure prediction mode).**

The results of A1 and A2 *TP53* intron 3 secondary structure predictions are shown in Table A7 (Additional file 1). According to our analyses, both structures with G-quadruplex (Figure 4 and Figure 5) have lower free energy than the respective canonical structures (Figure 6 and Figure 7), thus, the G-quadruplex structure would be preferred. However, the differences in free energy between the canonical structures of both polymorphic variants are significantly higher than between the respective G-quadruplexes. Finally, the predicted free energy is lower for the longer variant (A2) in both structures (canonical or G-quadruplex), which may account for its greater *in vivo* stability.

## Discussion

The differences in the sequencing of *TP53* between DNA and cDNA (mRNA) may result from the nonequivalence of the alleles’ impact on expression due to specific polymorphisms or epigenetic changes [14]. To investigate the hypothetical role of PIN3 polymorphism in *TP53* expression, we compared the frequencies of both polymorphic variants between the cases with and without the mentioned differences. Secondly, we analyzed which PIN3 variant is the mutated one in cases showing such discrepancies and conducted a reporter assay to compare the expression levels of both variants. Finally, we performed a bioinformatic analysis of the stability of both alleles with respect to their potential structures (canonical or G-quadruplex). The mutational analysis showed that among the 45 cases with a *TP53* mutation, the majority exhibited differences between DNA and cDNA sequencing (66.7%). The group without such discrepancies consisted mostly of A1/A1 homozygotes (86.7%), of one A2/A2 homozygote and one heterozygote. The other group also comprised mainly A1/A1 homozygotes (63.3%), but the frequency of heterozygotes was higher (6 cases – 20.0%) and there were 5 cases of A2/A2 homozygotes (16.7%). Such a distribution suggests a relation of the longer variant with the differences between the expression of both alleles. Since the appropriate PIN3 analysis required 3 criteria (PIN3 A1/A2 heterozygosity, codon 72 C/G heterozygosity and DNA/cDNA differences), it could be performed in only 6 out of 250 tumor cases. In all A1/A2 heterozygotes with the discussed DNA/cDNA differences the longer variant (A2) was the mutated one. This observation may support the hypothesis that mutations within the allele demonstrating a higher expression result in the differences in the sequencing patterns. Conversely, Gemignani *et al.* showed that the shorter allele (A1) should be related to the higher expression of *TP53* [14], however, their analyses were based on immortalized normal lymphocyte cells, known to present domains of monoallelic expression, which are possibly artifactual [26], and which may insufficiently reflect the conditions within tumor cells irrespective of the cell origin [14]. The *TP53* gene is most frequently mutated in solid tumors [27] – the highest percentage of mutations in this study was detected in colorectal cancer (19/31; 61.3%) and glioma (25/97; 25.8%), in hematological malignancies these mutations are less frequent (in this study only 8.7% samples diagnosed with acute myeloid leukemia were mutated), but often strongly correlated with unfavorable prognosis and resistance to therapy [28, 29].

Next, we performed a dual luciferase reporter analysis to test the hypothetical influence of specific PIN3 variants on *TP53* expression. Its results support the association of the A2 variant with the higher mRNA expression in comparison to the A1 variant. This observation is especially important in the light of the TP53 tetramer structure, whose proper function is possible only with all wild-type subunits [30], therefore the increased expression of the mutated allele will further abolish the activity of the wild-type TP53. PIN3 may be a potential explanation of the differences between DNA and cDNA analysis in cases with PIN3 heterozygosity. However, it may only apply to a minority of cases showing *TP53* haploinsufficiency, as the frequency of PIN3 heterozygosity is estimated as 20% in European population [14], 25% in South America and 31% in Asia [31, 32]. As reported in several case–control studies, PIN3 A2 allele is associated with an increased risk of various cancer types, particularly in colorectal and breast cancer (only in heterozygotes in case of the latter) [13–15, 33].

Finally, we took a closer look at the sequence of intron 3. It has a relatively small size (of 93 bp), therefore, the additional 16 bp insertion leads to an increase of intron length by 17%. Such change can lead to alteration in protein function or gene expression [12, 16–18]. Furthermore, the duplicated fragment consists of series of three or four guanines which are potentially able to form secondary structures – G-quadruplexes. Such structures within intron 3 of *TP53* pre-mRNA were confirmed by Marcel *et al.* [12]. G-quadruplexes on DNA strands function as regulators of replication and transcription. These motifs are especially common in the regions upstream of transcription start site of regulatory genes or oncogenes, while rarely within tumor suppressor genes [34], which advocates for the significance of PIN3. Since the duplication both significantly increases the intron's length and contains additional G-tracts, it most probably affects the topology of the G-quadruplexes and its stability, which, in turn, may have an impact on the transcript and, subsequently, on *TP53* expression level. G-quadruplexes within pre-mRNA have already been confirmed [12], therefore, it was necessary to inquire whether such structures can be formed within DNA. A preliminary bioinformatic analysis showed that both DNA variants are capable of forming G-quadruplexes, however, with varied stability. The predicted free energy of the longer variant (A2) was lower, therefore, the G-quadruplex structure would be more stable (Table A7). The impact of G-quadruplex on transcription depends on its location. G-quadruplex structures within the template strand inhibit transcription, whereas those within the non-template strand enhance the process [35]. G-tracts can also participate in hybrid quadruplexes (HQ) formation, which are intermolecular forms of G-quadruplexes formed between non-template DNA and nascent mRNA [36]. Undoubtedly, the HQ structures require more attention due to their significant role in the regulation of transcription, both *in vitro* and *in vivo* [36].

## Conclusions

The presented data strongly suggest that the *TP53* allele with PIN3 duplication shows higher expression of *TP53* mRNA in comparison to the allele without the duplication. A single mutation of the allele with PIN3 duplication in PIN3 heterozygotes (A1/A2) might be partially responsible for TP53 haploinsufficiency. These findings may provide a new insight into the search for the unknown haploinsufficiency mechanism and further therapeutic applications.

## Electronic supplementary material

Additional file 1: Table A1: The sequences of primers used for PCR and sequencing analysis of *TP53* exons. **Table A2.** The primer sequences used for *TP53* intron 3 sequencing. **Table A3.** Sequences of primers used during PIN3 and codon 72 bacterial subcloning procedure. **Table A4.** Results from dual luciferase reporter assays. **Table A5.** Intron 3 sequence from *TP53* gene, potentially capable of forming G-quadruplex structure. **Table A6.** Summary results of *TP53* sequencing analysis from 307 samples with the distinction for the diagnosis. **Table A7.** The results of RNAfold prediction. (PDF 318 KB)
