# Supplementary material for: PIN3 duplication may be partially responsible for TP53 haploinsufficiency
Source: BMC Cancer. 2014 Sep 15;14:669. doi: 10.1186/1471-2407-14-669 (PMC4176858; doi:10.1186/1471-2407-14-669)
Supplement: Supplementary file 1 — Additional file 1: Table A1: The sequences of primers used for PCR and sequencing analysis of TP53 exons. Table A2. The primer sequences used for TP53 intron 3 sequencing. Table A3. Sequences of primers used during PIN3 and codon 72 bacterial subcloning procedure. Table A4. Results from dual luciferase reporter assays. Table A5. Intron 3 sequence from TP53 gene, potentially capable of forming G-quadruplex structure. Table A6. Summary results of TP53 sequencing analysis from 307 samples with the distinction for the diagnosis. Table A7. The results of RNAfold prediction. (PDF 318 KB) [file 12885_2014_4860_MOESM1_ESM.pdf]

Additional files

Table A1. The sequences of primers used for PCR and sequencing analysis of *TP53* exons.

**Region**	**Sequences of primers**	
	**Forward**	**Reverse**
	**Polymerase chain reaction**	
**Exons 2-3**	5’-atccccacttttcctcttgc-3’	5’-gaaaagagcagtcagaggac-3’
**Exon 4**	5’-cctctgactgctcttttcacc-3’	5’-tcatggaagccagcccctcag-3’
**Exon 5**	5’-ttccacacccccgcccggca-3’	5’-accctgggcaaccagccctg-3’
**Exon 6**	5’-acagggctggttgcccaggg-3’	5’-agttgcaaaccagacctcag-3’
**Exon 7**	5’-actggcctcatcttgggcct-3’	5’-gtcagaggcaagcagaggct-3’
**Exon 8**	5’-taaatgggacaggtaggacc-3’	5’-tccaccgcttcttgtcctgc-3’
**cDNA exons 4-8**	5’-gagccgcagtcagatcctag-3’	5’-gcagtgctcgcttagtgctc-3’
	**Sequencing analysis**	
**Exons 2-3**	5’-tcttgcagcagccagact-3’	5’-agagcagtcagaggaccag-3’
**Exon4**	5’-ccatctacagtcccccttgc-3’	5’-caactttgggacaggagtcag-3’
**Exon 5**	5’-caagcagtcacagcacatga-3’	5’-aaccagccctgtcgtctct-3’
**Exon 6**	5’-caggcctctgattcctcact-3’	5’-agacctcaggcggctcatag-3’
**Exon 7**	5’-atctcctaggttggctctga-3’	5’-tggcaagtggctcctgacct-3’
**Exon 8**	5’-ctcttttcctatcctgagta-3’	5’-ctgcttgcttaccctgctta-3’
**cDNA exons 4-8**	5’-ccctctgagtcaggaaacat-3’	5’-ccctttcttgcggagattct-3’

Table A2. The primer sequences used for *TP53* intron 3 sequencing.

**Region**	**PCR Primer**	**Sequencing Primer**
**Intron 3**	FV 5’-atccccacttttcctcttgc-3’<br>REV 5’-tcatggaagccagcccctcag-3’	FV 5’-tcttgcagcagccagact-3’<br>REV 5’-caactttgggacaggagtcag-3’

Table A3. Sequences of primers used during PIN3 and codon 72 bacterial subcloning procedure.

**Type of reaction**		**Sequences of primers**
**Cloning**	BamHI restriction enzyme	5’-gcgaaggatccatgggactgactttctgctc-3’
	HindIII restriction enzyme	5’-cctggaagcttttctgggaagggacagaaga-3’
**Polymerase chain reaction**	Forward	5’-taaaacgacggccagtgaat-3’
	Reverse	5’-caggaaacagctatgaccatga-3’
**Sequencing analysis**	Forward	5’-gactgactttctgctcttgt-3’
	Reverse	5’-gctatgaccatgattacgcc-3’

Table A4. Results from dual luciferase reporter assays.

**Normalized Firefly luciferase activity**			
**Cell line**	**Construct**	**Normalized luciferase activity**	**Mean value**
AD293	C	0.265; 0.370; 0.517; 0.294; 0.288; 0.311; 0.260; 0.232; 0.254; 0.128; 0.195	0.283
	A1	0.481; 0.467; 0.669; 0.350; 0.432; 0.405; 0.306; 0.315; 0.285; 0.150; 0.267	0.375
	A2	0.544; 0.561; 0.773; 0.385; 0.476; 0.446; 0.344; 0.332; 0.317; 0.189; 0.365	0.430
SW962	C	0.023; 0.036; 0.036; 0.044; 0.037; 0.045; 0.044; 0.037	0.038
	A1	0.022; 0.047; 0.035; 0.040; 0.042; 0.036; 0.034; 0.038	0.037
	A2	0.038; 0.082; 0.056; 0.053; 0.077; 0.054; 0.048; 0.060	0.059
**Normalized luciferase activity in reference to control sample**			
**Cell line**	**Ratio**	**Ratio value**	**Mean value**
AD293	A1/C	1.82; 1.26; 1.29; 1.19; 1.50; 1.30; 1.18; 1.36; 1.12; 1.17; 1.37	1.32
	A2/C	2.05; 1.52; 1.50; 1.31; 1.65; 1.43; 1.32; 1.43; 1.25; 1.48; 1.87	1.53
	A2/A1	1.13; 1.20; 1.16; 1.10; 1.10; 1.10; 1.12; 1.05; 1.11; 1.26; 1.37	1.16
SW962	A1/C	0.96; 1.31; 0.97; 0.91; 1.14; 0.80; 0.77; 1.03	0.98
	A2/C	1.65; 2.28; 1.56; 1.20; 2.08; 1.20; 1.09; 1.62	1.59
	A2/A1	1.73; 1.74; 1.60; 1.33; 1.83; 1.50; 1.41; 1.58	1.59
**Results of statistical analysis**			
**Cell line**	**Ratio**	**p value**	
AD293	A1 vs. C	0.05566	
	A2 vs. C	0.00833	
	A1 vs. A2	0.33164	
	A1/C vs. A2/C	0.01923	
SW962	A1 vs. C	0.64538	
	A2 vs. C	0.00109	
	A1 vs. A2	0.00109	
	A1/C vs. A2/C	0.00186	
Total	A1/C vs. A2/C	0.00019	

Table A5. Intron 3 sequence from *TP53* gene, potentially capable of forming G-quadruplex structure [9].

TP53 intron 3 (A1)	GTAAGGACAAGGGTTGGGCTGGGGACCTGGAGGGCTGGGG GGCTGGGGGGCTGAGGACCTGGTCCTCTGACTGCTCTTTTC ACCCATCTACAG
TP53 intron 3 (A2)	GTAAGGACAAGGGTTGGGCTGGGGACCTGGAGGGCTGGGG ACCTGGAGGGCTGGGGGGCTGGGGGGCTGAGGACCTGGTC CTCTGACTGCTCTTTTCACCCATCTACAG

Table A6. Summary results of *TP53* sequencing analysis from 307 samples with the distinction for the diagnosis[2,3,19,20].

Diagnosis	Samples with *TP53* mutation	Samples without *TP53* mutation	Total number of samples	% of mutated samples
**glioma**	25	72	97	25.8%
**soft tissue sarcoma**	9	85	94	9.6%
**colorectal cancer**	19	12	31	61,3%
**prostate cancer**	1	19	20	5%
**leukemia (AML)**	2	21	23	8.7%
**invasive breast duct carcinoma**	8	34	42	19.0%

Table A7. The results of RNAfold prediction.

	Canonical structure prediction mode		G-quadruplex prediction mode	
	A1	A2	A1	A2
Minimum free energy kcal/mol	-9.60	-11.90	-23.16	-25.36
	GTAAGGACAAGGGTTGGGCTGGGGACCTGGAGGGCTGGGGGGCTGGGGGGCTGAGGACCTGGTCCTCTGACTGCTCTTTTCACCCATCTACAG<br>.......(((...)))..(((.(((..........((((.((..(((.((..((((((...))))))...)).)))...)).))))))).)))	GTAAGGACAAGGGTTGGGCTGGGGACCTGGAGGGCTGGGGACCTGGAGGGCTGGGGGGCTGGGGGGCTGAGGACCTGGTCCTCTGACTGCTCTTTTCACCCATCTA<br>CAG<br>.......(((...)))......((.(((..(((........)))..))).))(((.((..(((.((..((((((...))))))...)).)))...)).))).....<br>...	GTAAGGACAAGGGTTGGGCTGGGGACCTGGAGGGCTGGGGGGCTGGGGGGCTGAGGACCTGGTCCTCTGACTGCTCTTTTCACCCATCTACAG<br>..........+++..+++..+++........+++...++.++..++.++...((((((...))))))..........................	GTAAGGACAAGGGTTGGGCTGGGGACCTGGAGGGCTGGGGACCTGGAGGGCTGGGGGGCTGGGGGGCTGAGGACCTGGTCCTCTGACTGCTCTTTTCACCCATCTA<br>CAG<br>.........(((...+++..+++........+++..+++..))).........++.++..++.++...((((((...))))))......................<br>...
